# Relative Validity and Reproducibility of a Food-Frequency Questionnaire for Estimating Food Intakes among Flemish Preschoolers

**DOI:** 10.3390/ijerph6010382

**Published:** 2009-01-22

**Authors:** Inge Huybrechts, Guy De Backer, Dirk De Bacquer, Lea Maes, Stefaan De Henauw

**Affiliations:** 1 Department of Public Health, Ghent University, University Hospital 2BlokA, De Pintelaan 185, 9000 Gent, Belgium; E-Mails: guy.debacker@ugent.be (G. DB.); lea.maes@ugent.be (L. M.); dirk.debacquer@ugent.be (D. DB.); 2 Department of Health Sciences, Vesalius, Hogeschool Gent, Keramiekstraat 80, 9000 Gent, Belgium; E-Mail: stefaan.dehenauw@ugent.be (S. DH.)

**Keywords:** Relative validity, reproducibility, FFQ, food intake, preschool, children, Belgium

## Abstract

The aims of this study were to assess the relative validity and reproducibility of a semi-quantitative food-frequency questionnaire (FFQ) applied in a large region-wide survey among 2.5–6.5 year-old children for estimating food group intakes. Parents/guardians were used as a proxy. Estimated diet records (3d) were used as reference method and reproducibility was measured by repeated FFQ administrations five weeks apart. In total 650 children were included in the validity analyses and 124 in the reproducibility analyses. Comparing median FFQ1 to FFQ2 intakes, almost all evaluated food groups showed median differences within a range of ± 15%. However, for median vegetables, fruit and cheese intake, FFQ1 was > 20% higher than FFQ2. For most foods a moderate correlation (0.5–0.7) was obtained between FFQ1 and FFQ2. For cheese, sugared drinks and fruit juice intakes correlations were even > 0.7. For median differences between the 3d EDR and the FFQ, six food groups (potatoes & grains; vegetables Fruit; cheese; meat, game, poultry and fish; and sugared drinks) gave a difference > 20%. The largest corrected correlations (>0.6) were found for the intake of potatoes and grains, fruit, milk products, cheese, sugared drinks, and fruit juice, while the lowest correlations (<0.4) for bread and meat products. The proportion of subjects classified within one quartile (in the same/adjacent category) by FFQ and EDR ranged from 67% (for meat products) to 88% (for fruit juice). Extreme misclassification into the opposite quartiles was for all food groups < 10%. The results indicate that our newly developed FFQ gives reproducible estimates of food group intake. Overall, moderate levels of relative validity were observed for estimates of food group intake.

## 1. Introduction

An adequate diet is of profound importance in early childhood. To optimize childrens’ diets, knowledge about their actual intake must be obtained. Although the assessment of nutrient intakes can provide important information on dietary adequacy, intervention trials such as the CARET study showed that whole foods rather than individual nutrients may best indicate the potential role of the diet in disease prevention [1]. Hence, there is an increasing need for reliable measurements of foods that are consumed as part of the usual diet. However, accurate assessment of food intakes of free-living persons and especially of children remains a difficult and labour-intensive process. No single assessment method of an individual’s usual intake is optimal under all conditions. The choice of method depends, for instance, on the aim of the study, the skills of the study population, the accuracy of the dietary data required, and the funds and personnel available [2]. One of the most accurate methods to calculate dietary intake is the weighted food record. However, this method is time consuming and generally suitable only for individuals or small groups of cooperative volunteers [2]. Routine assessment of diet in a large number of individuals from a range of socioeconomic backgrounds requires a quicker and simpler method for estimating the intake of specific nutrients. Food-frequency questionnaires (FFQ) are shown to be a practical and efficient approach to assess habitual diet over periods of time and are widely used as cost-effective dietary assessment methods in large-scale dietary surveys to investigate customary food intakes over extended periods of time [2, 3]. Therefore, a new semi-quantitative FFQ was developed for use in the Flanders preschool dietary survey, to estimate calcium and food (group) intakes among Flemish preschoolers. Although reproducibility and relative validity of this newly developed FFQ for estimating preschoolers’ calcium intakes have been reported before [4], the reproducibility and validity of this FFQ for estimating preschoolers’ food intake is still unknown. Although validation studies for estimating food intakes among young children using parentally reported FFQ’s are rather scars [5, 6], like all dietary assessment methods, estimates derived from FFQ data suffer from random and systematic error and may not represent the ‘true’ usual diet. Numerous factors may compromise the validity of food consumption estimates [2, 7]. Therefore, in this study, the relative validity of food intake estimates derived from the FFQ that was administered by the parents or another caregiver in the Flanders preschool dietary survey is evaluated by comparison with the 3d estimated diet record (EDR). Those food intakes derived from both instruments can then both be compared with the Flemish Food Based Dietary Guidelines (FBDG) (Table 1).

## 2. Subjects and Methods

Data used for these analyses derived from a cross-sectional study among preschool children (2.5–6.5 y old) in Flanders, using a multistage clustered sampling design, with schools as primary sampling units (PSU) and classes as secondary sampling units. The study design and methodology of this study have been described in more detail previously [8]. In brief, a general questionnaire and a 47-item semi-quantitative FFQ (about the past year) were completed by an adult or proxy who spent most time with the child (usually the mother). In addition, 3d estimated dietary records (EDR) were collected about one week after the collection of the completed FFQs.

The data from the 3d EDR were used to calculate mean and median daily nutrient intakes per child. The frequency categories used in the semi-quantitative FFQ were: every day; 5–6 days per week; 2–4 days per week; 1 day per week and 1–3 days per month. The food categories in the FFQ were based on the classification system described in the Flemish food guide [9]. As an additional objective was to estimate calcium intake of Flemish preschool children, food(group)s with a high calcium content and part of the typical Flemish diet or with a moderate calcium content but commonly eaten by children were also included in the FFQ. The usual food intakes derived from the FFQ were calculated by multiplying the frequency of consumption with the daily portion size for each food group. More details about the dietary assessment instruments used have been published by Huybrechts *et al.* [4, 8].

In total 650 preschool children from the Flanders preschool dietary survey completed a FFQ and a good quality 3d EDR and could therefore be used for these relative validity analyses of the FFQ.

For the reproducibility study, 244 children have been selected in a separate sample of three nursery schools in the province ‘East-Flanders’. In total, 169 subjects returned a FFQ during the first administration, of whom 124 returned a second FFQ too. There was a time span of at least 5 weeks between the two FFQ administrations. A detailed description of the methodology used in this validation and reproducibility study has been reported by Huybrechts *et al.* [8].

### 2.1. Statistical Analysis

The distributions of most food group intakes were not normally distributed. Therefore, non-parametric methods were used in the reproducibility and relative validity analysis. Different statistical methods were used to evaluate reproducibility and relative validity of the FFQ.

The reproducibility between the first and second FFQ administration was estimated by means of the Wilcoxon signed-rank test, the Spearman rank order correlation coefficients and the intra-class correlations.

The validity of FFQ relative to the reference method (3d EDR) was assessed by the Wilcoxon signed-rank test, the Spearman rank order correlation, the weighted kappa (κ) statistic and misclassification analyses.

The correlation coefficients were also corrected for attenuation due to random error in the reference measurements as described by Liu *et al.* [10]. The degree of misclassification was estimated by examining the proportion of subjects classified by the reference method that fell into the same, into the adjacent, and into the extreme quartile when classified by the FFQ. Misclassification into the extreme quartile comprises both misclassifications from the first to the fourth quartile, and vice versa; from the fourth to the first quartile. Agreement has also been assessed using the weighted κ statistic, calculated with a linear set of weights [11].

Furthermore agreement between the EDR and the FFQ at an individual level was assessed using mean difference and standard deviation of the difference, which was visually shown in a Bland and Altman plot (Appendix 1) [12].

It is noteworthy that FFQs, in which less than half of the frequency questions had been answered, were excluded for all the analysis. Though, other FFQ, containing one or little missing values for frequency or portion size questions, remained included in the final database. However, because of missing values, those children could be excluded for some particular analyses, what explains the differences in number of subjects for the different analyses.

## 3. Results

### 3.1. Reproducibility Study

Table 2 shows the mean and median intakes of different food groups estimated from the repeated FFQ administrations and the results derived from the Wilcoxon signed-rank test. Comparing the mean intake of FFQ1 relative to FFQ2, almost all evaluated food groups showed mean differences within a range of ± 10%, indicating a high consistency in population estimates. Comparison of median intakes between the two administrations showed differences within a range of ± 15%. However, for median vegetables, fruit and cheese intake, the FFQ1 gave more than 20% higher values than FFQ2.

The Wilcoxon signed-rank test showed no statistically significant differences at the food group level between the intakes assessed at time one and time two. The mean and median intake of the second administration were for almost all food groups lower than or equal to the mean and median intake of the first administration.

Spearman correlations between both FFQ are presented in Table 3. For most foods a moderate correlation (0.5–0.7) was obtained. Though for cheese, sugared drinks and fruit juice intakes the spearman correlation was higher than 0.7, showing food correlations.

### 3.2. Relative Validity

Large differences in relative validity were found between the different food groups. From Table 4 it could be concluded that there was no overall tendency for the questionnaire measurements to give higher or lower means and medians than the reference method. Only six food groups gave mean differences within ± 10%, six food groups showed values that gave within 11–30% difference, and one food group (cheese) gave a difference > 40%. For median differences between the EDR and the FFQ, six food groups (potatoes & grains; vegetables Fruit; cheese; meat, game, poultry and fish; and sugared drinks) gave a difference of more than 20% of the EDR.

For six out of 13 food groups (beverages; potatoes and grains; vegetables; cheese; meat, game, poultry and fish; and fruit juice), the intake distribution of the FFQ was significantly different from the EDR intake distribution (<0.01) (table 4). However, it should be noted that comparison of the food group intakes reported by the two dietary assessment methods with the FBDG shown in table 1 are giving similar conclusions, except from the food groups meat products and cheese, for which the mean FFQ intakes were lower than the recommended levels, while mean EDR intakes complied with the recommendations.

Except from beverages (excl. milk products and drinks from restgroup), milk products, sugared drinks, and fruit juices and fried potatoes, the ratio of within- over between-subject variation measured in the 3d EDR was >1 for all other food groups. The within- over between-subject variation ranged from −16.25 for fried potatoes to 9.87 for potatoes and grains (Table 5). This lack of precision in the reference measurements (EDR) was taken into account by computing deattenuated correlation coefficients. These corrected Spearman correlations between the FFQ and the 3d EDR are presented in Table 5. The largest corrected Spearman correlations (>0.6) were found for the intake of potatoes and grains, fruit, milk products, cheese, sugared drinks, and fruit juice, while the lowest correlations (<0.4) were found for bread products and meat products (Table 5).

The degree of misclassification associated with categorized intakes assessed by the FFQ was examined as the proportion of subjects classified into the same, the adjacent, or opposite quartile (Table 6). The proportion of subjects classified within one quartile (this is in the same/adjacent category) by both instruments ranged from 67% (for meat products) to 88% (for fruit juice). Extreme misclassification into the opposite quartiles was for all food groups less than 10%. The highest value was seen for meat products and snacks, 9% and 8% respectively. Findings from the weighted κ statistic showed moderate agreement (0.40–0.50) for milk products and fruit juices and poor agreement (<0.20) for bread and cereals, potatoes and grains, meat products and snacks (Table 6).

Graphical observation of the Bland and Altman plots for the different food groups showed for many foods increasing bias of the food intakes, estimated by the FFQ with increasing mean daily intakes (Appendix 1).

## 4. Discussion

### 4.1. Main results and Comparison with Other Studies

Although this study showed good reproducibility for almost all food groups, large differences in relative validity were found between the different food groups. The largest percentage of mean difference was found for cheese intake for which the mean intake calculated from the EDR was almost twice as large as the mean intake calculated from the FFQ. Though, despite those important differences in mean food group intakes derived from the two different methods, similar conclusions can be drawn from both methods when comparing the mean intakes with the food-based dietary guidelines (FBDG) (presented in table 1). So, for measuring lacunas in the preschool dietary habits, both instruments could give a similar rough estimation of the major gaps.

However, from the misclassification analysis it was obvious that for different food groups this FFQ and EDR could not equally discriminate between quartiles. Also findings from the weighted κ statistic showed poor to moderate agreement for most of the food groups.

Corrected Spearman correlations between the FFQ and the 3d EDR showed values between 0.32 and 0.75. Other validation studies of intake of food groups and single food items assessed by FFQ have observed correlations, generally between 0.3 and 0.8 [2, 13–16].

Although the usefulness and validity of this FFQ for estimating an effect/change in dietary habits over time should be further investigated, from the good reliability (reproducibility) of the FFQ and the moderate to good correlations between the FFQ and the 3d EDR, it could be presumed that this FFQ might be a useful instrument for measuring effect/change in dietary habits among preschool children in future intervention studies.

### 4.2. Strengths and Weaknesses of the Study

The EDR was chosen as reference method because of its high level of accuracy when validated for assessing dietary intake in infants and children [17]. Moreover, the measurement errors of the EDR and the FFQ are highly independent, since unlike the FFQ method the EDR does not depend on memory, is open-ended, and involves direct estimation of portion size [16]. However, like any dietary assessment methodology, the EDR is prone to a degree of misreporting.

For instance, **Day-to-day variability** in preschoolers’ diets might be responsible for some larger differences between the results derived from the FFQ and the EDR. The low within- over between-individual variability ratio of milk products implies that most preschool children in Flanders are consuming milk products on a regularly (daily) basis. The high variance ratios for the other food groups however are due to the large day-to-day variability in food consumption. For the food groups showing high variance ratios, the reference (3d EDR) measurements may be biased and imperfectly reflect ranking. Therefore, Spearman correlations were corrected for attenuation, which improved the correlations for all food groups.

It is noteworthy that the low values for agreements between the FFQ and the 3d EDR might also be due to the important within-person variability for some of the food groups derived from the 3d EDR.

Even though many recording days (replicates) should give a better estimate of the habitual intake, the problems with long recording periods are declining accuracy of recording with increasing fatigue and boredom, and potential alterations to dietary habits [18]. Because of those disadvantages of long recording periods and in the hope to minimize the refusal rate and/or drop-out within the study, it was decided to use 3d EDR in this validation study. However, a higher number of record days, spread over the whole year would have been more optimal as reference method, since this could take into account seasonal variation as well.

In addition, difficulties **in portion size** estimations during completion of the 3d EDR might also bias the true validity of the FFQ. For food groups, which are difficult to quantify in standard or household units in the EDRs (e.g. vegetables, meat, potatoes, rice, etc. which are often part of a mixed dish) the difference could be significant between the FFQ and the EDR results. In the 3-day EDR, for food groups like vegetables, meat products and potatoes, the dieticians coding the EDR had to assign standard portion sizes when the respondent was not able to quantify the consumed amount of food in grams (e.g. during school lunches). Since no standard portion sizes were available for children in Belgium, those from the general Belgian population had to be used instead [19]. It should be noted that these standard portion sizes could have been too high for children and consequently have introduced non-negligible differences between the FFQ and EDR-results. In addition, a standard portion size, often had to be used for ‘a slice of cheese’ [19]. However, the authors presume that the weight used for a slice of cheese, [19] which was also borrowed from the manual of our general Belgian population might have been too high for preschool children, which could explain the higher cheese intake estimated from the EDRs.

As described in more depth previously [8], like most surveys, our sample of preschool children included in the relative validity analyses was subject to some non-participation bias, in which higher social classes are likely to be over-represented.

A limitation of the reproducibility study could be a possible memory effect during completion of the second FFQ as parents could possibly still remember what they filled in five weeks ago.

Although differences in relative validity and reproducibility between different age categories might exist, the validity and reproducibility analyses were only performed on the total sample of preschool children (between 2.5 and 6.5 years old), as power would be too low for the reproducibility analyses if two different age groups were considered.

At last it should be noted that because of the lack of an external marker, no validity analyses could be performed for the reference method (3-d EDR) that was used in this validation study. Although biochemical measurements of nutrient and contaminant levels in blood or other body tissues/fluids can provide a useful assessment of the intake of certain nutrients or contaminants (especially for those that are measured poorly by other methods in children), it should be noted that children remain a special case with different limitations in the area of biochemical samples as well [20, 21].

The major disadvantage to biomarkers collected via blood samples is the fact that the invasive nature of venipuncture puts some limits on researchers ability to obtain samples from children or to get high participation rates in large-scale studies. Also urine collections in young children for whom urine collection procedures require special consideration is not always easy. Appropriate sample collection apparatus, such as urine collection bags or toilet inserts, must be provided to collect urine samples from children who are not yet completely toilet trained (e.g. still wearing nappies at night) [21]. Because of those constraints related to the use of biomarkers in childhood populations, no external markers have been used in the current study.

### 4.3. Recommendations

When developing a FFQ, it is important to weigh carefully the pro’s and contra’s of asking more food items within the FFQ. Usually compromises need to be made between respondent burden and the level of detail required. For the food groups showing low validity and/or reproducibility, additional analyses could be recommended to investigate whether further disaggregating or aggregating of some food groups could be recommended. Therefore, the data derived from the 3d EDR collected in the Flanders preschool dietary survey can be analyzed by performing stepwise regression analysis in order to select the additional food groups that should be added to the FFQ [22, 23]. However, food groups that were under consumed by this preschool population (e.g. coffee and thee) might be excluded from the food list when the respondent burden would be compromised by the disaggregating of some other food groups.

## 5. Conclusions

The results indicate that this newly developed FFQ gives reproducible estimates of food group intakes. Though, large day-to-day variation in food group intake of the 3d EDR data complicated the evaluation of FFQ relative validity. Overall, moderate levels of relative validity were observed for estimates of food group intakes. Therefore, from the reproducibility and validity analyses executed so far, we could conclude that the FFQ developed for use in the Flanders preschool dietary survey can be used for ranking subjects according to their calcium [4] and food group intakes. In addition, the low respondent burden and logistical implications of the brief FFQ could make it a useful tool for doing trend analyses. Though, stepwise regression analyses on the 3d EDR data could be used to optimize the FFQ.

## Figures and Tables

**Table 1:** Food based dietary guidelines (FBDG).

Food group	FBDG
Beverages (total)*	1000 mL
Bread & cereals	90–150 g
Potatoes & grains (no crisps)	50–200 g
Vegetables (no juices & soups)	100–150 g
Fruit (no juices)	125–250 g
Milk products^μ^	500 mL
Cheese	10–20 g
Meat, game, poultry and fish	75–100 g
Restgroup (snacks)^†^	Restricted
Restgroup (sugared drinks)^‡^	Restricted
Fruit juice	Moderate
Restgroup (fried potatoes)	Restricted
Restgroup (sweet spreads)	Restricted

* All drinks (incl. juices, but no milk products and no drinks from restgroup).

μ Milk, sugared milk drinks, yoghurt, milk deserts, and calcium enriched soy drinks.

† Restgroup (snacks): sweet deserts (e.g. ice cream, chocolate mousse), sweet snacks, salty snacks (e.g. chips), chocolate and brioches.

‡ Restgroup (sugared drinks): sugared drinks (e.g. tea with sugar added) and soft drinks, but no fruit juices.

**Table 2:** Comparison of mean and median food (group) intakes estimated from the first and the second FFQ in the reproducibility study.

Food group	N	Mean (g/d)		Median (g/d)		SD (g/d)		Mean Δ	Sign.#
		FFQ1	FFQ2	FFQ1	FFQ2	FFQ1	FFQ2		
Beverages (total)*	105	575	536	515	500	291	248	39	0.090
Bread & cereals	111	78	78	84	82	36	37	0	0.921
Potatoes & grains (no crisps)	112	138	136	140	140	58	60	2	0.642
Vegetables (no juices & soups)	^113^	82	73	94	58	45	47	9	0.059
Fruit (no juices)	103	119	118	150	119	60	62	1	0.710
Milk productsμ	96	416	386	381	364	217	199	30	0.103
Cheese	115	9	9	9	6	8	9	0	0.854
Meat, game, poultry and fish	106	69	66	66	65	25	22	3	0.166
Restgroup (snacks)†	103	56	54	47	51	40	34	2	0.585
Restgroup (sugared drinks)‡	113	93	86	22	22	166	136	7	0.608
Fruit juice	115	198	184	150	150	167	148	14	0.226
Restgroup (fried potatoes)	118	12	13	14	14	9	10	−1	0.538
Restgroup (sweet spreads)	108	12	13	10	11	9	10	−1	0.626

^#^ Wilcoxon signed-rank test

* All drinks (incl. juices, but no milk products and no drinks from restgroup)

^μ^ Milk, sugared milk drinks, yoghurt, milk deserts, and calcium enriched soy drinks

^†^ Restgroup (snacks): sweet deserts (e.g. ice cream, chocolate mousse), sweet snacks, salty snacks (e.g. chips), chocolate, and brioches.

^‡^ Restgroup (sugared drinks): sugared drinks (e.g. tea with sugar added) and soft drinks, but no fruit juices.

N (124)

**Table 3:** Spearman correlations and intra-class correlations (ICC) between FFQ1 and FFQ2 for food(group) intakes.

Food group	N	Spearman	ICC
Beverages (total)*	105	0.60	0.65
Bread & cereals	111	0.58	0.54
Potatoes & grains (no crisps)	112	0.64	0.61
Vegetables (no juices & soups)	113	0.56	0.58
Fruit (no juices)	103	0.66	0.68
Milk productsμ	96	0.70	0.70
Cheese	115	0.80	0.79
Meat, game, poultry and fish	106	0.62	0.59
Restgroup (snacks)†	103	0.53	0.53
Restgroup (sugared drinks)‡	113	0.86	0.79
Fruit juice	115	0.74	0.62
Restgroup (fried potatoes)	118	0.61	0.60
Restgroup (sweet spreads)	108	0.51	0.53

* All drinks. (incl. juices, but no milk products and no drinks from restgroup)

^μ^ Milk, sugared milk drinks, yoghurt, milk deserts, and calcium enriched soy drinks.

^†^ Restgroup (snacks): sweet deserts (e.g. ice cream, chocolate mousse), sweet snacks, salty snacks (e.g. chips), chocolate, and brioches.

^‡^ Restgroup (sugared drinks): sugared drinks (e.g. tea with sugar added) and soft drinks, but no fruit juices.

N (124)

**Table 4:** Comparison of mean and median food (group) intakes estimated from the FFQ with mean and median intakes derived from the EDR.

Food group	N	Mean		Median		SD		Mean Δ	Sign. #
		FFQ	EDR	FFQ	EDR	FFQ	EDR		
Beverages (total)*	619	521	489	504	458	245.6	261	32	0.001
Bread & cereals	636	87	93	86	89	36.5	42	−6	0.052
Potatoes & grains (no crisps)	634	131	101	121	96	62.9	54	30	<0.001
Vegetables (no juices & soups)	632	80	66	85	57	46.4	46	14	<0.001
Fruit (no juices)	634	112	109	118	92	63.8	87	3	0.021
Milk productsμ	605	422	446	405	425	235	243	−24	0.022
Cheese	638	8	14	6	8	9	17	−6	<0.001
Meat, game, poultry and fish	616	72	91	69	87	28	41	−19	<0.001
Restgroup (snacks)†	618	58	58	54	51	35	34	0	0.856
Restgroup (sugared drinks)‡	633	88	99	22	50	130	146	−11	0.077
Fruit juice	642	151	174	118	133	143	182	−23	0.002
Restgroup (fried potatoes)	639	13	15	14	0	10	21	−2	0.893
Restgroup (sweet spreads)	637	16	15	11	12	13	13	1	0.015

^#^ Wilcoxon signed-ranks test.

* All drinks. (incl. juices, but no milk products and no drinks from restgroup)

^μ^ Milk, sugared milk drinks, yoghurt, milk deserts, and calcium enriched soy drinks.

^†^ Restgroup (snacks): sweet deserts (e.g. ice cream, chocolate mousse), sweet snacks, salty snacks (e.g. chips), chocolate, and brioches.

^‡^ Restgroup (sugared drinks): sugared drinks (e.g. tea with sugar added) and soft drinks, but no fruit juices.

N (661)

**Table 5:** Spearman correlations between FFQ and EDR for food (group) intakes.

Food group	N	Spearman	Variance Ratio	Corrected correlation
Beverages (total)*	619	0.503	0.76	0.563
Bread & cereals	636	0.297	1.15	0.349
Potatoes & grains (no crisps)	629	0.299	9.87	0.619
Vegetables (no juices & soups)	632	0.384	2.99	0.542
Fruit (no juices)	634	0.541	1.50	0.662
Milk productsμ	605	0.598	0.57	0.653
Cheese	638	0.540	2.82	0.752
Meat, game, poultry and fish	616	0.234	2.72	0.323
Restgroup (snacks)†	618	0.245	6.42	0.434
Restgroup (sugared drinks)‡	633	0.569	0.72	0.633
Fruit juice	633	0.616	0.68	0.682
Restgroup (fried potatoes)	633	0.247	−16.25	NA
Restgroup (sweet spreads)	633	0.435	1.86	0.554

* All drinks (incl. juices, but no milk products and no drinks from restgroup) NA = not applicable

^μ^ Milk, sugared milk drinks, yoghurt, milk deserts, and calcium enriched soy drinks

^†^ Restgroup (snacks): sweet deserts (e.g. ice cream, chocolate mousse), sweet snacks, salty snacks (e.g. chips), chocolate, and brioches.

^‡^ Restgroup (sugared drinks): sugared drinks (e.g. tea with sugar added) and soft drinks, but no fruit juices.

N (661)

**Table 6:** Comparison of FFQ with mean daily intakes derived from 3d EDR, based on joint classification by quartiles.

Food group	FFQ versus 3d EDR			Weighted κ
	Same category (%)	Adjacent category (%)	Extreme category (%)	
Beverages (total)*	39	39	4	0.33
Bread & cereals	31	41	6	0.18
Potatoes & grains (no crisps)	31	40	8	0.17
Vegetables (no juices & soups)	35	40	5	0.25
Fruit (no juices)	41	41	2	0.36
Milk productsμ	43	38	2	0.43
Cheese	NA	NA	NA	NA
Meat, game, poultry and fish	32	35	9	0.15
Restgroup (snacks)†	32	40	8	0.19
Restgroup (sugared drinks)‡	NA	NA	NA	NA
Fruit juice	44	44	2	0.42
Restgroup (fried potatoes)	NA	NA	NA	NA
Restgroup (sweet spreads)	39	38	4	0.31

* All drinks (incl. juices, but no milk products and no drinks from restgroup)

^μ^ Milk, sugared milk drinks, yoghurt, milk deserts, and calcium enriched soy drinks

^†^ Restgroup (snacks): sweet deserts (e.g. ice cream, chocolate mousse), sweet snacks, salty snacks (e.g. chips), chocolate, and brioches.

^‡^ Restgroup (sugared drinks): sugared drinks (e.g. tea with sugar added) and soft drinks, but no fruit juices.

NA: for the food groups cheese, fried potatoes, and sugared drinks, no quartiles could be calculated since

>25% of the children did not consume any cheese or sugared drinks during the 3d EDR period.

## Appendix 1

Bland and Altman plots of major food groups for visualizing differences between the mean food group intakes of the 3d EDR and the FFQ.

## References

[1] Neuhouser ML, Patterson RE, Thornquist MD, Omenn GS, King IB, Goodman GE (2003). Fruits and vegetables are associated with lower lung cancer risk only in the placebo arm of the beta-carotene and retinol efficacy trial (CARET). Cancer Epidemiol. Biomarkers Prev.

[2] Willett WC (1998). Nutritional Epidemiology.

[3] Treiber FA, Leonard SB, Frank G, Musante L, Davis H, Strong WB, Levy M (1990). Dietary assessment instruments for preschool children: reliability of parental responses to the 24-hour recall and a food frequency questionnaire. J. Am. Diet. Assoc.

[4] Huybrechts I, de Bacquer D, Matthys C, de Backer G, de Henauw S (2006). Validity and reproducibility of a semi-quantitative food-frequency questionnaire for estimating calcium intake in Belgian preschool children. Br. J. Nutr.

[5] Livingstone MB, Robson PJ (2000). Measurement of dietary intake in children. Proc. Nutr. Soc.

[6] Livingstone MB, Robson PJ, Wallace JM (2004). Issues in dietary intake assessment of children and adolescents. Br. J. Nutr.

[7] Block G, Hartman AM (1989). Issues in reproducibility and validity of dietary studies. Am. J. Clin. Nutr.

[8] Huybrechts I, Matthys C, Pynaert I, de Maeyer M, Bellemans M, de Geeter H, de Henauw S (2008). Flanders preschool dietary survey: rationale, aims, design, methodology and population characteristics. Arch. Public Health.

[9] VIG De voedingsdriehoek: een praktische voedingsgids 2004.

[10] Liu K, Stamler J, Dyer A, McKeever J, McKeever P (1978). Statistical methods to assess and minimize the role of intra-individual variability in obscuring the relationship between dietary lipids and serum cholesterol. J. Chronic. Dis.

[11] Altman DG (1991). Practical Statistics for Medical Research.

[12] Bland JM, Altman DG (1986). Statistical methods for assessing agreement between two methods of clinical measurement. Lancet.

[13] Bohlscheid-Thomas S, Hoting I, Boeing H, Wahrendorf J (1997). Reproducibility and relative validity of food group intake in a food frequency questionnaire developed for the German part of the EPIC project. European Prospective Investigation into Cancer and Nutrition. Int. J. Epidemiol.

[14] Feskanich D, Rimm EB, Giovannucci EL, Colditz GA, Stampfer MJ, Litin LB, Willett WC (1993). Reproducibility and validity of food intake measurements from a semiquantitative food frequency questionnaire. J. Am. Diet. Assoc.

[15] Salvini S, Hunter DJ, Sampson L, Stampfer MJ, Colditz GA, Rosner B, Willett WC (1989). Food-based validation of a dietary questionnaire: the effects of week- to-week variation in food consumption. Int. J. Epidemiol.

[16] Cade J, Thompson R, Burley V, Warm D (2002). Development, validation and utilisation of food-frequency questionnaires - a review. Public Health Nutr.

[17] Lanigan JA, Wells JC, Lawson MS, Lucas A (2001). Validation of food diary method for assessment of dietary energy and macronutrient intake in infants and children aged 6–24 months. Eur. J. Clin. Nutr.

[18] Gibson RS (1987). Sources of error and variability in dietary assessment methods: A review. J. Can. Dietet. Assoc.

[19] de Backer G (2005). Maten en Gewichten. Handleiding voor gestandaardiseerde kwantificering van voedingsmiddelen in België, revisie januari 2005.

[20] Holland NT, Smith MT, Eskenazi B, Bastaki M (2003). Biological sample collection and processing for molecular epidemiological studies. Mutat. Res.

[21] Wessels D, Barr DB, Mendola P (2003). Use of biomarkers to indicate exposure of children to organophosphate pesticides: implications for a longitudinal study of children’s environmental health. Environ. Health Perspect.

[22] Shahar D, Fraser D, Shai I, Vardi H (2003). Development of a food frequency questionnaire (FFQ) for an elderly population based on a population survey. J. Nutr.

[23] Shai I, Shahar DR, Vardi H, Fraser D (2004). Selection of food items for inclusion in a newly developed food-frequency questionnaire. Public Health Nutr.

